# Prognostic Value of Preoperative Lymphocyte-to-Monocyte Ratio in Patients with Recurrent Colorectal Cancer

**DOI:** 10.3390/medicina61040707

**Published:** 2025-04-11

**Authors:** Oğuzcan Özkan, Pınar Peker, Aslı Geçgel, Erdem Göker

**Affiliations:** 1 Department of Medical Oncology, Ege University Faculty of Medicine, 35040 Izmir, Turkey; dr.asltrgt@gmail.com (A.G.); erdem.goker@ege.edu.tr (E.G.); 2 Department of Medical Oncology, Adana State Hospital, 01170 Adana, Turkey; pnnarrrr@gmail.com

**Keywords:** colorectal cancer, lymphocyte-to-monocyte ratio, prognosis, recurrence, survival

## Abstract

*Background and Objectives*: CRC is one of the leading causes of cancer-related deaths worldwide. New biomarkers are needed to identify the high-risk patient population after primary treatment and to personalize and perfect treatment and follow-up. Indicators of cancer-associated systemic inflammatory response, such as the LMR, have been widely investigated and have yielded conflicting results. The aim of this study was to investigate the effect of preoperative LMR on the prognosis of recurrent CRC. *Materials and Methods*: We included 204 patients admitted to our center for recurrent CRC between January 2010 and January 2015. Retrospectively, we investigated the preoperative LMR data and its effect on RFS and OS. *Results*: The cut-off value of LMR was 24.72 and, according to this value, we created two groups: LMR-H and LMR-L. There were 104 (50.9%) patients in the H group and 100 (49.1%) patients in the L group. The median OS was 38.0 months (95% confidence interval (CI): 30.66–45.33) for the L group and 49.0 months (95% CI: 44.06–53.94) for the H group. Overall population median OS was calculated as 44.0 months (95% CI: 40.1–47.8, *p* = 0.004). Median RFS was 21.3 months (95% CI: 18.3–24.2) for the LMR-L group and 28.39 months (95% CI: 24.9–31.8) for the LMR-H group (*p* = 0.004). *Conclusions*: The association between the LMR at diagnosis and early recurrence, as well as survival outcomes, was investigated in patients with recurrent CRC. Higher preoperative LMR levels were found to correlate with improved OS and RFS.

## 1. Introduction

CRC is a prevalent malignancy worldwide, ranking as the third most commonly diagnosed cancer and the second leading cause of cancer-related death, contributing significantly to the global health burden and healthcare costs [1]. Epidemiological studies reveal an increasing trend in CRC incidence, particularly in younger populations in high-income countries, due to factors such as changes in lifestyle, diet, and obesity rates [2,3]. Early-stage CRC is often asymptomatic or presents with nonspecific symptoms, leading to delayed diagnosis and poorer prognoses in many cases. Current standard CRC diagnostic approaches primarily rely on colonoscopy and histopathological biopsy, while treatment regimens include a combination of surgery, endoscopic treatments, chemotherapy, radiotherapy, and more recently, immunotherapy [4,5,6]. Despite advancements in these therapies, the prognosis for CRC remains challenging, especially in cases of recurrence and advanced stages where survival rates decrease significantly [7].

Early detection, diagnosis, and treatment have been shown to significantly improve outcomes in CRC. Known prognostic factors such as tumor depth, lymph node metastasis, and TNM staging play a critical role in determining CRC patient prognosis [8]. However, even among patients with similar TNM stages and tumor grades, prognosis can vary widely, underscoring the need for additional prognostic markers that may help predict disease outcomes more accurately [9].

Inflammation has been recognized as a key component in carcinogenesis, as it contributes to various processes such as tumor promotion, progression, and metastasis. Chronic inflammation and immune dysregulation are now recognized as contributors to CRC development and progression, with increasing evidence supporting the prognostic role of systemic inflammatory markers in cancer patients [10,11,12]. Markers of systemic inflammation, particularly those based on hematological parameters, have gained considerable interest for their potential in prognostication. Ratios like neutrophil-to-lymphocyte (NLR), platelet-to-lymphocyte (PLR), and LMR have emerged as valuable indicators of systemic inflammation and immune response in CRC and other malignancies [13,14,15].

The LMR reflects a balance between the antitumor immune response (lymphocytes) and tumor-associated macrophage activity (monocytes), where a lower LMR has been associated with worse outcomes in several cancers. Studies indicate that high NLR and PLR, alongside low LMR, are often linked to poor prognosis and higher mortality rates in CRC patients [16,17,18]. Yet, the prognostic power of LMR has been less extensively studied in patients with recurrent CRC, leaving a gap in understanding its utility in this subset of patients who face a particularly challenging prognosis.

Thus, this study aims to evaluate the prognostic significance of LMR in patients with recurrent CRC who underwent surgical resection. By examining LMR as a potential predictive factor for OS and RFS in this population, we seek to provide further insight into how systemic inflammation markers could be used to stratify patients’ prognoses and guide clinical management in recurrent CRC.

## 2. Materials and Methods

### 2.1. Study Design and Patients

It was planned in 204 patients with recurrent CRC who were followed up and treated in our tertiary oncology clinic between January 2010 and January 2015. Approval for this study was obtained from the ethics committee of our university. All patients included in this analysis met the following criteria: patients with a complete pathological report after postoperative pathological diagnosis; those who did not use neoadjuvant CT, CRT and immunomodulatory agents before surgery; who did not have secondary malignancy; who had blood and routine examination results in the file system of the preoperative period; and those who had no rheumatological and infectious history that could affect inflammatory markers in the preoperative period. The entire population of these 204 patients consists of cases who had undergone curative treatment and were found to have recurrence during follow-up surveillance tests. In the detection of recurrence, additional tests such as carcinoembryonic antigen, conventional cross-sectional imaging, and colonoscopy were used.

Clinical and laboratory data were collected from medical records on the hospital information system. Laboratory data (lymphocyte and monocyte counts) were collected from each patient’s blood sample and blood tests were taken after fasting, in the morning before surgery.

### 2.2. Statistical Analysis Methods

ROC analysis and median LMR was applied to determine the appropriate cut-off value to predict the effect of LMR on prognosis. According to this cut-off value, patients were divided into ‘high’ (H group) and ‘low’ (L group) LMR groups. Baseline demographic and clinical characteristics between both groups were compared using chi-square and Fisher’s exact test. The effect of LMR on OS and RFS was evaluated using the Kaplan–Meier method and log-rank test. For the multivariate analysis, variables that showed significant differences in the univariate analysis were selected. In addition, multivariate Cox regression analysis was performed to determine independent prognostic factors. In this study, patients were censored according to standard guidelines. All statistical analyses were performed using SPSS software (version 23.0; IBM Corp., Armonk, NY, USA). *p* < 0.05 was accepted as the statistical significance criterion.

## 3. Results

### 3.1. Patient Characteristics

Of the 204 patients included in the study, 55.9% were male and 35.3% were over 65 years of age. Adenocarcinoma was the dominant histological subtype in 81.4% of the patients. The tumors were localized on the left side in 80.4% of patients. Lymphovascular invasion was detected in 60.3% and perineural invasion in 45.6%. After median analysis, the LMR cut-off value was determined and LMR-H and LMR-L groups were formed. LMR cut-off values are presented in Table 1. Median follow-up was 42.10 months. The characteristics of the patients at the time of diagnosis are summarized in Table 2.

### 3.2. LMR Cut-Off Value

**Figure 1:**
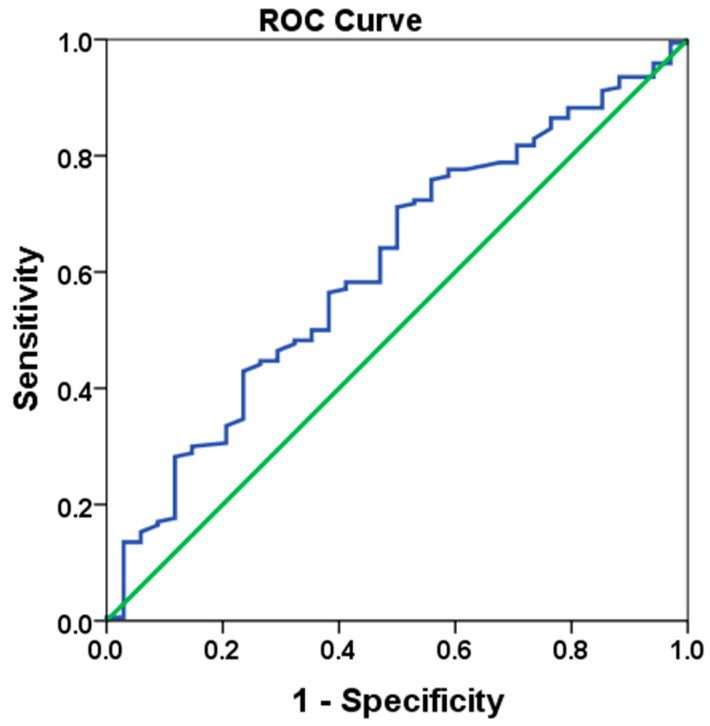
ROC curve analysis of LMR value (HR: 0.726, *p* = 0.041).

In this study, both ROC-derived and median cut-off values were utilized to assess the prognostic value of the LMR. The use of both methods allowed for a comparison of their performance in classifying patients and determining the most appropriate cut-off point. The cut-off derived from the ROC analysis was 25.96, with a sensitivity of 58.2% and specificity of 58.8% (See Figure 1). The Area Under the Curve (AUC) was 0.611 (95% CI: 0.509–0.712, standard error: 0.052, *p* = 0.042). These values are generally considered moderate to low, indicating that the ROC-derived cut-off may not be optimal for distinguishing between patient groups in terms of prognosis. Specifically, the low sensitivity and specificity suggest that the ROC-derived cut-off had limited accuracy in identifying patients with either high or low LMR values. This limitation is common in ROC analysis, especially when the AUC is low or when the chosen cut-off point does not effectively differentiate between the groups. The ROC curve determined to obtain the cut off LMR value is shown in Figure 1. Given these limitations, the median cut-off value of 24.72 was evaluated and found to perform better in patient classification. The median cut-off represents a more straightforward and robust method for categorizing patients, as it divides the population into two equal groups based on the central tendency of the data, thereby offering a more reliable classification. Therefore, patient groups were classified according to the median cut-off value rather than the ROC-derived value. This decision was made to provide more accurate prognostic stratification, as the median cut-off demonstrated superior performance in this analysis, despite the limitations observed with the ROC-derived cut-off. 

Patients with higher LMR according to the cut-off value were assigned to the H group and patients with lower LMR were assigned to the L group. There were 104 (50.9%) patients in the H group and 100 (49.1%) patients in the L group.

### 3.3. Comparison Between LMR-H and LMR-L Groups

Since the *p*-values for all clinical and demographic variables in this table are above 0.05, there is no statistically significant difference between the LMR-L and LMR-H groups for any variable. This indicates that LMR is not associated with these variables or that there is a homogeneous distribution between the groups.

### 3.4. OS Assessment Results by LMR Groups

**Figure 2:**
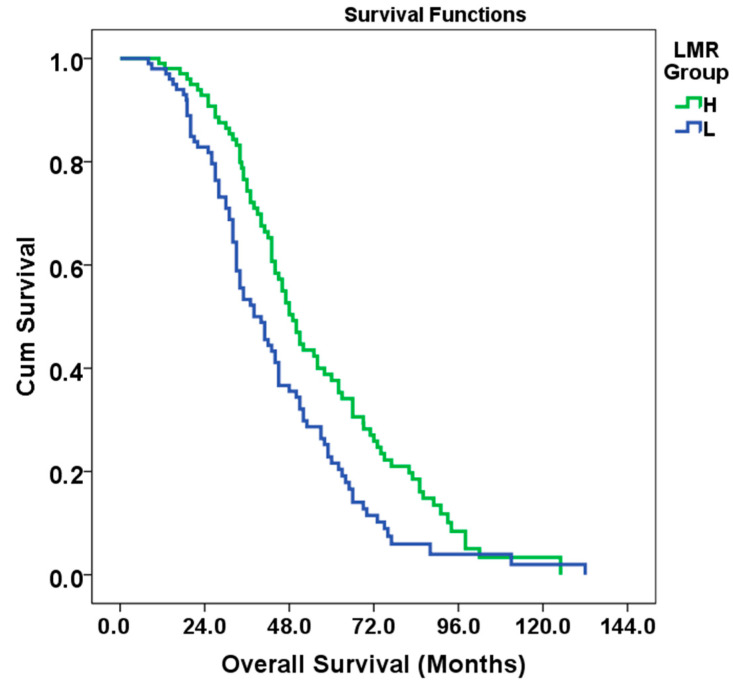
Kaplan–Meier curve of OS of patients classified according to LMR.

Patients were divided into two groups according to LMR as low LMR (L group) and high LMR (H group). In the survival time analysis, the median OS for the L group was 38 months (95% CI: 30.662–45.338, standard error: 2.5), while the median OS for the H group was 49 months (95% CI: 44.060–53.940, standard error: 2.8). The overall population median OS was 44 months (95% CI: 40.148–47.852, standard error: 1.9). Age, presence of RAS mutation, tumor grade, and nodal involvement were the most effective variables on survival. LVI was significant in univariate analysis but lost its significance in multivariate analysis. Univariate and multivariate OS analysis by subgroups is detailed in Table 3. In the multivariate analysis, a 34% reduction in the risk of death was observed in the high LMR group (95% CI:0.488–0.916, HR: 0.66, *p* = 0.012). The Kaplan–Meier curve of OS of patients classified according to LMR is given in Figure 2.

According to the results of the log-rank test (Mantel–Cox), the difference in survival between the LMR groups was statistically significant (Chi-square: 8.363, df: 1, *p* = 0.004) (See Figure 2).

### 3.5. Results of Evaluation of RFS According to LMR Groups

**Figure 3:**
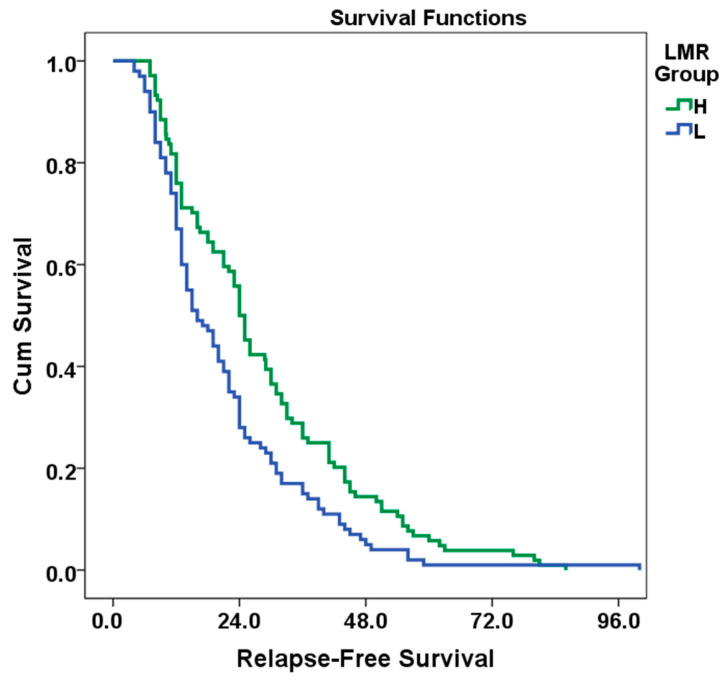
Kaplan–Meier curve of RFS of patients classified according to LMR.

In the survival analysis, the mean RFS for the L group was calculated as 21.3 months (95% CI: 18.317–24.283, standard error: 1.6), while the mean RFS for the H group was 28.3 months (95% CI: 24.909–31.888, standard error: 1.9, *p* = 0.004). The overall population mean RFS was 24.9 months (95% CI: 22.570–27.267, standard error: 1.3). These results show that there is a statistically significant relationship between LMR and RFS (*p* <0.05). In this case, the risk of relapse decreased by 32% in the high LMR group (95%CI: 0.518–0.909, HR: 0.68, *p* = 0.009). Kaplan–Meier curves of RFS according to LMR status are given in Figure 3. In univariate analysis, RAS mutation, tumor grade, T and N stage, LVI increased the risk of recurrence, while a high LMR value decreased the risk of recurrence. According to multivariate analysis, tumor grade, T and N stage variables increase the risk of recurrence. An increase in LMR is associated with a decrease in the risk of recurrence. RAS mutation and LVI status did not have a significant effect on the risk of recurrence, although it was very close to the significance limit in multivariate analysis. Analysis information is detailed in Table 4.

According to the results of the log-rank test (Mantel–Cox), the difference in survival between the LMR groups was statistically significant (Chi-square: 6.226, df: 1, *p* = 0.004).

## 4. Discussion

This study evaluates the prognostic value of the preoperative LMR in determining survival and risk of relapse in patients with recurrent CRC. The findings suggest that patients with higher LMR have longer OS and RFS, suggesting that this parameter may be a favorable prognostic factor for recurrence and survival. This finding is in line with a growing number of studies in the literature suggesting that LMR as an indicator of systemic inflammatory response has prognostic value in many solid tumors, including CRC [19]. Although previous studies have indicated that LMR has value as a prognostic indicator, there are few studies focusing on the recurrent CRC subgroup [16,20]. The present study is one of the rare studies evaluating LMR preoperatively in recurrent CRC patients and investigating the effect of this parameter on prognosis. Low LMR is generally associated with low lymphocyte and/or high monocyte levels, which may reflect a weak immune response and increased inflammatory response in cancer patients.

In our study, median OS was 38 months in patients with low LMR and 49 months in patients with high LMR. In the literature, a meta-analysis by Tan D. et al. shows that low LMR negatively affects survival in CRC patients [21]. Our results are supported by the study of Song W. et al. indicating that low preoperative LMR is associated with poor prognosis in patients with CRC. This suggests that it may be used as a marker reflecting the effect of the immune system on cancer progression [22].

The inflammatory response is also an important component of cancer progression, as it can not only destroy cancer cells but also create the tumor microenvironment that aids in the proliferation and metastasis of cancer cells [23]. Several studies in the literature have shown that various systemic inflammation factors such as PLR and NLR can be used to predict the prognosis of CRC patients [18,24,25]. It has also been demonstrated that a preoperative low LMR is associated with postoperative surgical complications [26]. We believe that LMR could be a potential new biomarker not only for CRC prognosis but also for predicting postoperative surgical complications. Some novel enzymatic biomarkers, such as butyrylcholinesterase, have also been studied in the perioperative period of CRC to investigate their effects on patient prognosis and postoperative complications [27]. Presumably, differences in tumor biology and tumor microenvironment may affect the immune response by influencing the severity of T cell disruption and the sensitivity of tumor cells to cytotoxic functions [28]. Lymphocyte count reflects the response of the host immune system. Lymphocytes inhibit the proliferation and spread of cancer [29]. Lymphopenia is often seen in advanced cancers and may result in a weak and inadequate immune response. Some studies have associated this with an unfavorable prognosis in oncological patients [30]. In contrast, monocytes can suppress the T cell response with the release of some anti-inflammatory cytokines and play a vital role in tumor progression [31]. A correlation between monocytosis and poor prognosis has been found in many cancers [30,32]. In summary, we think that this is one of the main mechanisms of the positive correlation between elevated LMR and CRC prognosis.

In our study, RAS mutation status, tumor grade, T and N stages, and LVI were found to increase the risk of relapse in univariate analysis, while only tumor grade and T and N stages remained significant in multivariate analysis. The fact that RAS mutation and lymphovascular invasion were not significant in multivariate analysis has been associated with different results in the literature. For example, some studies suggest that higher LMR value reduces tumor burden and is associated with less invasion, thus LVI may be indirectly related to LMR [33]. However, this relationship needs to be further investigated.

In our study, it was observed that high LMR levels reduced the risk of relapse by 32% (HR: 0.68, *p*= 0.009) and provided a longer RFS time compared to the low LMR group. The study by Guo G. et al. similarly shows that LMR can be used as a prognostic marker in the assessment of relapse risk. This suggests that suppression of inflammation and enhancement of immune response may reduce the risk of tumor progression and relapse [34]. As a biomarker of systemic inflammation, LMR has been proven to be a predictor for hematological malignancies and some solid cancers [35]. For example, a meta-analysis by Nishijima et al. showed that LMR is a prognostic factor for CRC patients [36]. The study by Shimura T. et al. in 2020 examined the prognostic impact of the LMR among CRC patients undergoing chemotherapy. The research revealed that a low LMR indicated that patients had worse RFS after treatment, and a value of LMR ≤ 2.4 was an independent prognostic factor [37]. Therefore, it was concluded that LMR plays an important role as a prognostic marker in CRC patients [37]. Some studies have suggested that LMR does not play a meaningful role as a prognostic marker in CRC patients or should be evaluated in combination with other biomarkers. These studies used different patient groups, stages, or treatment modalities, which may lead to different results. Different measurement methods or evaluation criteria may call into question the prognostic value of LMR. Evaluation of LMR in combination with other inflammatory or immunologic markers may provide more information [23,38]. For example, a study of locally advanced rectal cancer patients reported that LMR did not significantly predict poor prognostic predictors such as disease spread or extramural vascular invasion (EMVI). This suggests that the prognostic value of LMR in some patient subgroups may be limited or uncertain [39]. In another study, LMR was found to be associated with OS in patients with rectal cancer, but no significant association was found with tumor stage and other clinical factors. This suggests that LMR may not be sufficiently reliable as a prognostic indicator in some patient groups [40]. In one study, no significant correlation was found between peripheral blood count markers such as LMR and other inflammation markers and local inflammatory markers such as T-lymphocyte density in the tumor. This result suggests that blood-based markers such as LMR may not always be a direct indicator of the intra-tumor inflammatory response [41].

These studies suggest that LMR may not have prognostic value in some CRC patients or further studies are needed. This suggests that the prognostic value of LMR may vary according to patient characteristics and that more extensive research is required for its use as a definitive prognostic factor. Therefore, we performed the study to investigate the prognostic role of LMR in CRC patients.

The prognostic value of LMR may have an important role in clinical practice. Patients with low preoperative LMR may benefit from more frequent follow-up or aggressive treatment approaches. Furthermore, combining preoperative LMR values with other prognostic factors (age, tumor stage, treatment characteristics) to better assess the recurrence risk of patients may contribute to treatment decision-making. However, since our results are based on a retrospective cohort study, possible biases and limitations should be considered. It is important to confirm these findings with prospective, larger and multicenter studies.

In conclusion, the findings of our study suggest that high LMR values have positive effects on survival and relapse risk and that LMR can be used as a simple, inexpensive and feasible prognostic marker in CRC patients. In line with the literature, the evaluation of LMR together with other prognostic factors may contribute to clinical decision-making processes, especially in identifying patients at high risk of relapse. Obtaining more data with prospective, large-scale, and multicenter studies in this field will reveal the prognostic importance of LMR more strongly.

### Study Limitations

One of the main differences between our study and others is that all of our patients were cases of recurrent CRC, and we did not include a control group without recurrence. Regarding the limitations of our study, the small sample size and the fact that it was conducted at a single center are important factors that may limit the generalizability of our findings. Additionally, the retrospective design of the study introduces the potential for selection and information bias. We were also unable to obtain additional biochemical and pathological data from the patients, and the mismatch repair (MMR) status of the patients was not known. Furthermore, the lack of information regarding treatment plans after recurrence, comorbidities, and recurrence sites represents additional limitations. Another limitation is that the ROC-derived LMR cut-off value had low specificity and sensitivity, and, therefore, the median LMR value was used instead, which also introduces a limitation in our study. These factors should be considered when interpreting the results, and future studies should aim to address these issues, including a larger, multicenter cohort and a more comprehensive dataset.

## 5. Conclusions

Based on our findings, there is a significant relationship between immune-inflammatory indices and prognosis in CRC. Specifically, a higher LMR at diagnosis correlates positively with OS and RFS in CRC patients who experience recurrence. This suggests that a higher preoperative LMR may reflect a more robust immune response, which can contribute to improved long-term outcomes. Thus, LMR can serve as a valuable prognostic marker, aiding in risk stratification and potentially guiding treatment strategies for CRC patients. Further research into immune-inflammatory indices may enhance personalized care approaches in oncology.

## Figures and Tables

**Table 1:** ROC and median analysis values for LMR cut off value (with hazard ratio and *p* value).

	LMR Cut-off	Hazard Ratio (95% Cl)	*p*-Value
ROC	25.96	0.726 (0.535–0.986)	0.041
Median	24.72	0.638 (0.470–0.867)	0.004

**Table 2:** Clinicopathological characteristics of the patients at the time of diagnosis.

	Overall, n, %	LMR-L, n, %	LMR-H, n, %	*p*-Value
**Gender**				0.575
*Male*	114, 55.9	42, 42	48, 46.2	
*Female*	90, 44.1	58, 58	56, 53.8	
**Age(years)**				0.108
<*65*	132, 64.7	59, 59	73, 70.2	
≥*65*	72, 35.3	41, 41	31, 29.8	
**Histological subtype**				0.720
*Adenocarcinoma*	166, 81.4	80, 80	86, 82.7	
*Mucinous adenocarcinoma*	38, 18.6	20, 20	18, 17.3	
**Tumor location**				0.725
*Right-side*	40, 19.6	21, 21	19, 18.3	
*Left-side*	164, 80.4	79, 79	85, 81.7	
**RAS**				0.776
*Wild*	121, 59.3	58, 58	63, 60.6	
*Mutant*	83, 40.7	42, 42	41, 39.4	
**Histological grade**				0.938
*1*–*2*	187, 91.7	92, 92	95, 91.3	
≥*3*	17, 8.3	8, 8	9, 8.7	
**T-Stage**				0.655
*1*–*3*	138, 67.6	66, 66	72, 69.2	
≥*4*	66, 32.4	34, 34	32, 30.8	
**N Stage**				0.607
*Negative*	43, 21.1	23, 23	20, 19.2	
*Positive*	161, 78.9	77, 77	84, 80.8	
**LVI**				0.476
*No*	81, 39.7	37, 37	44, 42.3	
*Yes*	123, 60.3	63, 63	60, 57.7	
**PNI**				0.485
*No*	111, 54.4	57, 57	54, 51.9	
*Yes*	93, 45.6	43, 43	50, 48.1	

Abbreviations: LVI (Lymphovascular Invasion), PNI (Perineural Invasion), TNM (Tumor, Node, Metastasis).

**Table 3:** Univariate and multivariate OS analysis by subgroups.

	Univariate Analysis			Multivariate Analysis		
Variable	Hazard Ratio	95% Cl	*p* Value	Hazard Ratio	95% Cl	*p* Value
**Gender**	0.98	0.727–1.337	0.928			
*Male*						
*Female*						
**Age(years)**	1.45	1.060–1.997	0.020	1.57	1.127–2.199	0.008
<*65*						
≥*65*						
**Histological subtype**	0.69	0.472–1.024	0.066			
*Adenocarcinoma*						
*Mucinous adenocarcinoma*						
**Tumor location**	0.69	0.482–1.012	0.058			
*Right-side*						
*Left-side*						
**RAS**	1.43	1.054–1.944	0.022	1.46	1.070–1.992	0.017
*Wild*						
*Mutant*						
**Histological grade**	1.98	1.143–3.452	0.015	2.07	1.174–3.657	0.012
*1–2*						
*3*						
**T Stage**	1.34	0.970–1.850	0.076			
*1–3*						
*≥4*						
**N Stage**	1.52	1.039–2.239	0.031	1.65	1.104–2.481	0.015
*Negative*						
*Positive*						
**LVI**	1.42	1.037–1.951	0.029	1.30	0.947–1.808	0.103
*No*						
*Yes*						
**PNI**	1.34	0.985–1.824	0.062			
*No*						
*Yes*						
**LMR**	0.63	0.470–0.867	0.004	0.66	0.488–0.916	0.012
*H*						
*L*						

**Table 4:** Univariate and multivariate RFS analysis by subgroups.

	Univariate Analysis			Multivariate Analysis		
Variable	Hazard Ratio	95% Cl	*p* Value	Hazard Ratio	95% Cl	*p* Value
**Gender**	1.01	0.767–1.337	0.929			
*Male*						
*Female*						
**Age (years)**	1.27	0.950–1.699	0.107			
<*65*						
≥*65*						
**Histological subtype**	0.86	0.606–1.237	0.429			
*Adenocarcinoma*						
*Mucinous adenocarcinoma*						
**Tumor location**	0.79	0.559–1.121	0.188			
*Right-side*						
*Left-side*						
**RAS**	1.37	1.037–1.823	0.027	1.31	0.984–1.745	0.064
*Wild*						
*Mutant*						
**Histological grade**	2.16	1.309–3.594	0.003	1.81	1.073–3.067	0.026
*1*–*2*						
*3*						
**T Stage**	1.57	1.164–2.125	0.003	1.58	1.154–2.163	0.004
*1*–*3*						
≥*4*						
**N Stage**	1.49	1.058–2.120	0.023	1.51	1.061–2.173	0.022
*Negative*						
*Positive*						
**LVI**	1.39	1.046–1.848	0.023	1.26	0.942–1.695	0.118
*No*						
*Yes*						
**PNI**	1.24	0.939–1.645	0.128			
*No*						
*Yes*						
**LMR**	0.66	0.506–0.885	0.005	0.68	0.518–0.909	0.009
*H*						
*L*						

## Data Availability

The data used in the present study are available from the corresponding author upon request.
